# User-friendly extraction and multistage tandem mass spectrometry based analysis of lipid-linked oligosaccharides in microalgae

**DOI:** 10.1186/s13007-018-0374-8

**Published:** 2018-12-05

**Authors:** Pierre-Louis Lucas, Rodolphe Dumontier, Corinne Loutelier-Bourhis, Alain Mareck, Carlos Afonso, Patrice Lerouge, Narimane Mati-Baouche, Muriel Bardor

**Affiliations:** 1 UNIROUEN, Laboratoire Glyco-MEV EA4358, Normandie Univ, 76000 Rouen, France; 2 UNIROUEN, INSA Rouen, CNRS, COBRA, Normandie Univ, 76000 Rouen, France; 3 Institut Universitaire de France (IUF), 75000 Paris, France

**Keywords:** Diatom, *Chlamydomonas reinhardtii*, *Phaeodactylum tricornutum*, Lipid-linked oligosaccharides, Microalgae, Multistage tandem mass spectrometry

## Abstract

**Background:**

Protein *N*-glycosylation is initiated within the endoplasmic reticulum through the synthesis of a lipid-linked oligosaccharides (LLO) precursor. This precursor is then transferred *en bloc* on neo-synthesized proteins through the action of the oligosaccharyltransferase giving birth to glycoproteins. The *N*-linked glycans bore by the glycoproteins are then processed into oligomannosides prior to the exit of the glycoproteins from the endoplasmic reticulum and its entrance into the Golgi apparatus. In this compartment, the *N*-linked glycans are further maturated in complex type *N*-glycans. This process has been well studied in a lot of eukaryotes including higher plants. In contrast, little information regarding the LLO precursor and synthesis of *N*-linked glycans is available in microalgae.

**Methods:**

In this report, a user-friendly extraction method combining microsomal enrichment and solvent extractions followed by purification steps is described. This strategy is aiming to extract LLO precursor from microalgae. Then, the oligosaccharide moiety released from the extracted LLO were analyzed by multistage tandem mass spectrometry in two models of microalgae namely the green microalgae, *Chlamydomonas reinhardtii* and the diatom, *Phaeodactylum tricornutum.*

**Results:**

The validity of the developed method was confirmed by the analysis of the oligosaccharide structures released from the LLO of two xylosyltransferase mutants of *C. reinhardtii* confirming that this green microalga synthesizes a linear Glc_3_Man_5_GlcNAc_2_ identical to the one of the wild-type cells. In contrast, the analysis of the oligosaccharide released from the LLO of the diatom *P. tricornutum* demonstrated for the first time a Glc_2_Man_9_GlcNAc_2_ structure.

**Conclusion:**

The method described in this article allows the fast, non-radioactive and reliable multistage tandem mass spectrometry characterization of oligosaccharides released from LLO of microalgae including the ones belonging to the Phaeodactylaceae and Chlorophyceae classes, respectively. The method is fully adaptable for extracting and characterizing the LLO oligosaccharide moiety from microalgae belonging to other phyla.

**Electronic supplementary material:**

The online version of this article (10.1186/s13007-018-0374-8) contains supplementary material, which is available to authorized users.

## Background

*N*-glycosylation is a protein post-translational modification playing many roles in biological process [1]. For example, interactions between host and pathogens or between cells involve specific recognition between oligosaccharides and lectins [1]. Moreover, *N*-glycosylation ensures the conformation, the stability and the biological function of glycoproteins [2]. In plants, *N*-glycosylation has been widely studied, mainly in *Arabidopsis thaliana* and *Nicotiana benthamiana* [3–7]. The *N*-glycan synthesis starts in the endoplasmic reticulum (ER) with a two steps processing involving the action of a set of specific enzymes named Asparagine-Linked Glycosylation (ALG) [8]. The first step, beginning in the cytoplasmic side of the ER, results in the assembly of an oligosaccharide on a lipid carrier called dolichol pyrophosphate (PP-Dol) by successive additions of two *N*-acetylglucosamine and five mannose residues respectively through the action of ALG7, 13, 14, 1, 2 and 11. The lipid-linked oligosaccharide precursor (LLO) is then flipped towards the luminal side of the ER where the ALG3, 9 and 12 add four additional mannose residues, then respective actions of the ALG6, 8 and 10 elongate the LLO structure with three terminal glucose residues [9]. Such assembly of the LLO leads to the Glc_3_Man_9_GlcNAc_2_-PP-Dol precursor in plants which is identical to the structures identified in other eukaryotes [8, 9]. The second step involves the transfer of the oligosaccharide part of the LLO from the PP-Dol to an asparagine residue of a neosynthesized protein by the oligosaccharyltransferase complex (OST) [10, 11]. This is directly followed by the quality control of the protein folding involving, the removal of the three terminal glucose residues as a final step, leading to the formation of oligomannosidic *N*-linked glycans [12, 13]. This nascent folded glycoprotein is then transferred to the Golgi apparatus where *N*-linked glycans will undergo specific maturation by glycosyltransferases and glycosidases which are different among species and lead to different complex-type *N*-glycans. In plants, the *N*-glycosylation maturation process generates complex-type *N*-glycans GlcNAc_2_Man_3_GlcNAc_2_ bearing alpha(1,3)-core fucose and beta(1,2)-core xylose and eventually Lewis a epitopes [7, 14, 15].

So far, little attention has been paid to protein *N*-glycosylation in microalgae. Indeed, all the published work focused on the analysis of the *N*-glycan structures of four main microalgae: *Phaeodactylum tricornutum, Botryococcus braunii, Chlamydomonas reinhardtii and Porphyridium sp.* [16–20]. Major differences in complex *N*-glycan structures of these microalgae were recently reviewed [21]. For example, complex *N*-glycans containing a xylose moiety have been identified in *C. reinhardtii, Porphyridium sp. and Botryococcus braunii* but not in *P. tricornutum*. Currently, no functional study characterizing the enzymes involved in the LLO synthesis in microalgae is available in the literature. Preliminary bioinformatic genome analyses indicated that some ALG could be missing. Indeed, neither ALG3, ALG9 and ALG12 in *C. reinhardtii* nor ALG10 in both *P. tricornutum* and *C. reinhardtii* have been identified so far [16, 18, 22]. Because those genes are crucial for the complete synthesis of the Glc_3_Man_9_GlcNAc_2_ oligosaccharide, the lack of these steps could severely impact the synthesis of the final LLO precursor [9]. To the best of our knowledge, only one publication reported so far the structure of the LLO precursor from *C. reinhardtii* wild-type [23], demonstrating that *C. reinhardtii* accumulates a predominant linear truncated Glc_3_Man_5_GlcNAc_2_ LLO in the ER. Such a truncated structure probably results from the absence of the ALG genes as already described in protozoan parasites. Indeed, parasite LLO extracted from *Cryptosporidium parvum* and *Toxoplasma gondii* are characterized by the synthesis of a truncated single arm precursor with five mannose residues and two or three glucose residues [24, 25]. These studies tend to demonstrate that the LLO synthesis is not so well preserved in eukaryotes and deserves to be studied in other species like microalgae. Moreover, information regarding the functional property of LLO in microalgae is currently missing.

In this article, an user-friendly extraction method and multistage tandem mass spectrometry were combined to characterize oligosaccharides released from the LLO of microalgae. This method is elaborated from previous reported works in plants and parasites and has been validated on two xylosyltransferase mutants of the microalga *C. reinhardtii* which were compared to the wild-type cells. The oligosaccharide moiety released from the *C. reinhardtii* mutant LLO was demonstrated to correspond to a linear Glc_3_Man_5_GlcNAc_2_ structure confirming previous results [23]. Then, the protocol has been applied to the Pt1.8.6 strain of the diatom *P. tricornutum* in order to determine the structure of its LLO-released oligosaccharide. Such results demonstrate the adaptability of the method described herein for further studies of LLO extracted from other microalgae.

## Results and discussion

### Optimization of a protocol for the LLO precursor extraction and characterization in microalgae

Nowadays, most of the protocols reported in the literature for LLO extractions and analyses are based on radioactivity labelling. Cells are incubated with radioactive ^3^H- or ^14^C-labelled metabolic precursors which are incorporated into LLO through the cell metabolism [26–28]. This strategy allows detection and analysis of low quantity of LLO but requires specific health and safety practices and cannot be performed in all laboratories due to the hazard management. Moreover, as incubation times with radioactive precursors are often very short, the incorporated radioactivity may not reflect the complete set of oligosaccharides produced by the organisms and the labelled material represents minute amount which is not compatible with a detailed structural analysis of the radioactive compounds.

**Fig. 1:**
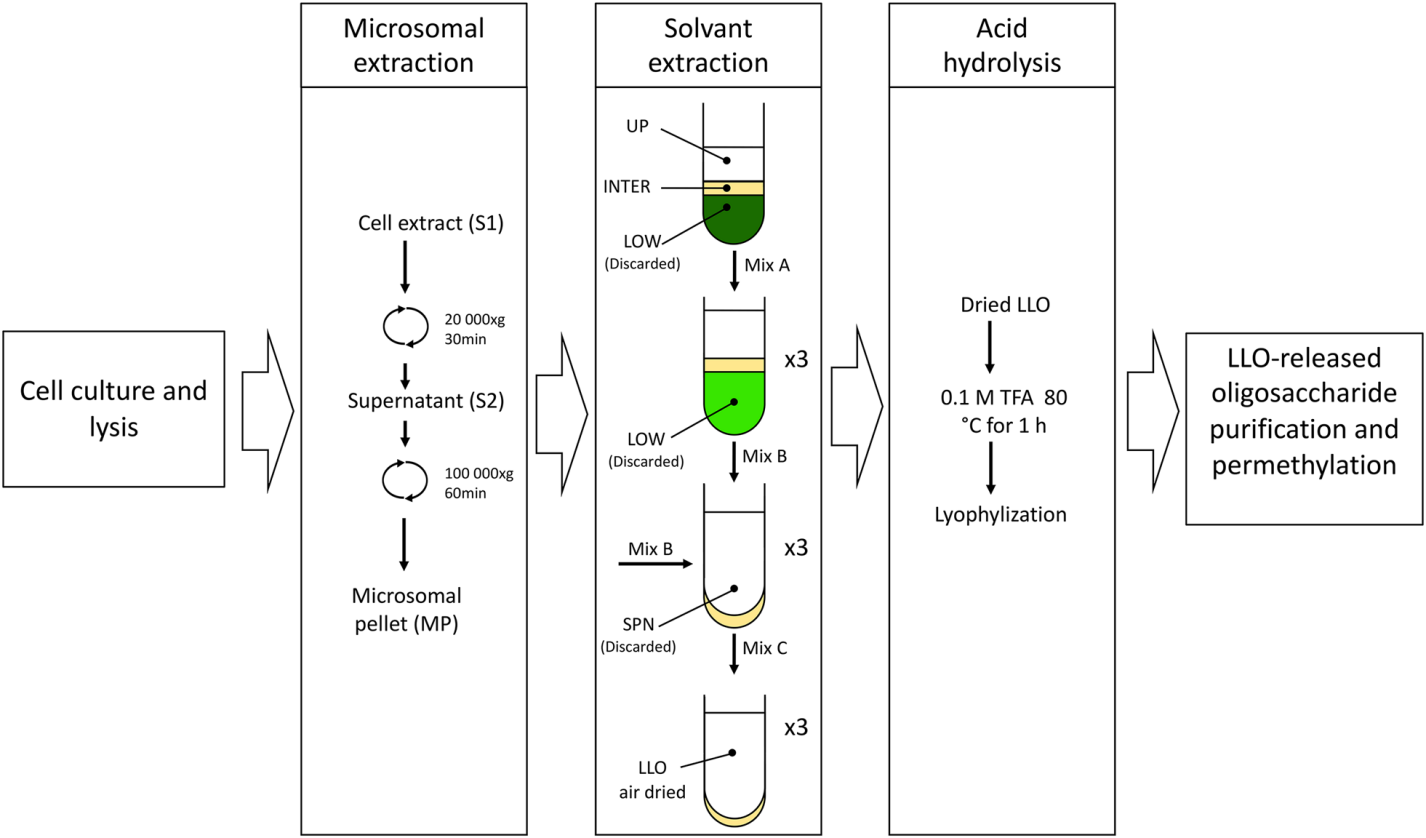
Scheme summarizing the main steps of the Lipid-Linked Oligosaccharide (LLO) extraction method used in this study to extract and purify the oligosaccharide released from LLO of microalgae. UP: upper phase; INTER: intermediate phase; LOW: lower phase; SPN: supernatant resulting from solvent extraction, Mix: mixture; S: supernatant resulting from centrifugation; MP: microsomal pellet

Here, we developed a simple protocol for LLO extraction without any radioactive labelling. In this purpose, the LLO were optimally extracted by the combination of two methods previously reported for *T. gondii* and *A. thaliana,* respectively [24, 29]. The first step of the protocol consisted in cell homogenization and a centrifugation step at 10 000xg. The cell homogenization was optimized for *C. reinhardtii* and *P. tricornutum* respectively as their cell wall composition is completely different. The diatom cells containing a more rigid cell wall require more grinding cycles for efficient cell lysis. Therefore, in future application, we would recommend for other microalgae species to apply either the specie-specific cell lysis protocol if available or the one used herein for *P. tricornutum*. It is to note that our extraction procedure does not include lipase inhibitors. However, the PMSF used during the cell lysis step might help decreasing lipase activity as previously described [30, 31]. Therefore in future application, if low LLO-released oligosaccharides are recovered using the protocol described here, it may be worth adding lipase inhibitors before cell breakage to make sure that lipase activities are not degrading the linkage between the dolichol phosphate and the oligosaccharide. Then, the microsomal fraction containing the LLO anchored in the ER were recovered from the other cell components by successive centrifugation steps at 20,000×*g* followed by an ultracentrifugation step at 100,000×*g*. The resulting pellet corresponding to the enriched microsomal fraction was then collected (MP in Fig. 1). The second step corresponds to the LLO purification using successive solvent extraction procedures. Finally, two different methods have been tested in order to hydrolyze the pyrophosphate bond between the lipid and the oligosaccharide of the LLO. The first one consisted in resuspending the LLO in 0.01 M HCl in tetrahydrofuran and incubated the sample at 50 °C for 2 h [24]. The second method involved the hydrolysis of the LLO using 0.1 M of trifluoroacetic acid (TFA) at 80 °C for 1 h [29]. In our hands, only the mild acidic hydrolysis using 0.1 M of TFA gave sufficient amount of LLO-released oligosaccharide for further multistage tandem mass spectrometry analysis. Figure 1 summarizes the overall user-friendly protocol of LLO extraction in microalgae. After purification of the LLO-released oligosaccharide using a Carbograph column, the oligosaccharide was permethylated as previously described [32]. Permethylation of samples presents two main analytical advantages. Firstly, permethylated oligosaccharides are less polar than their native forms and so are more efficiently ionized in MALDI and ESI [33, 34]. Secondly, this greatly facilitates the discrimination between glycan isomers when permethylated oligosaccharides are fragmented by collision-induced dissociation multistage tandem mass spectrometry [35–37].

### Identification of oligosaccharide isolated from the LLO of the green microalgae *Chlamydomonas reinhardtii*

**Fig. 2:**
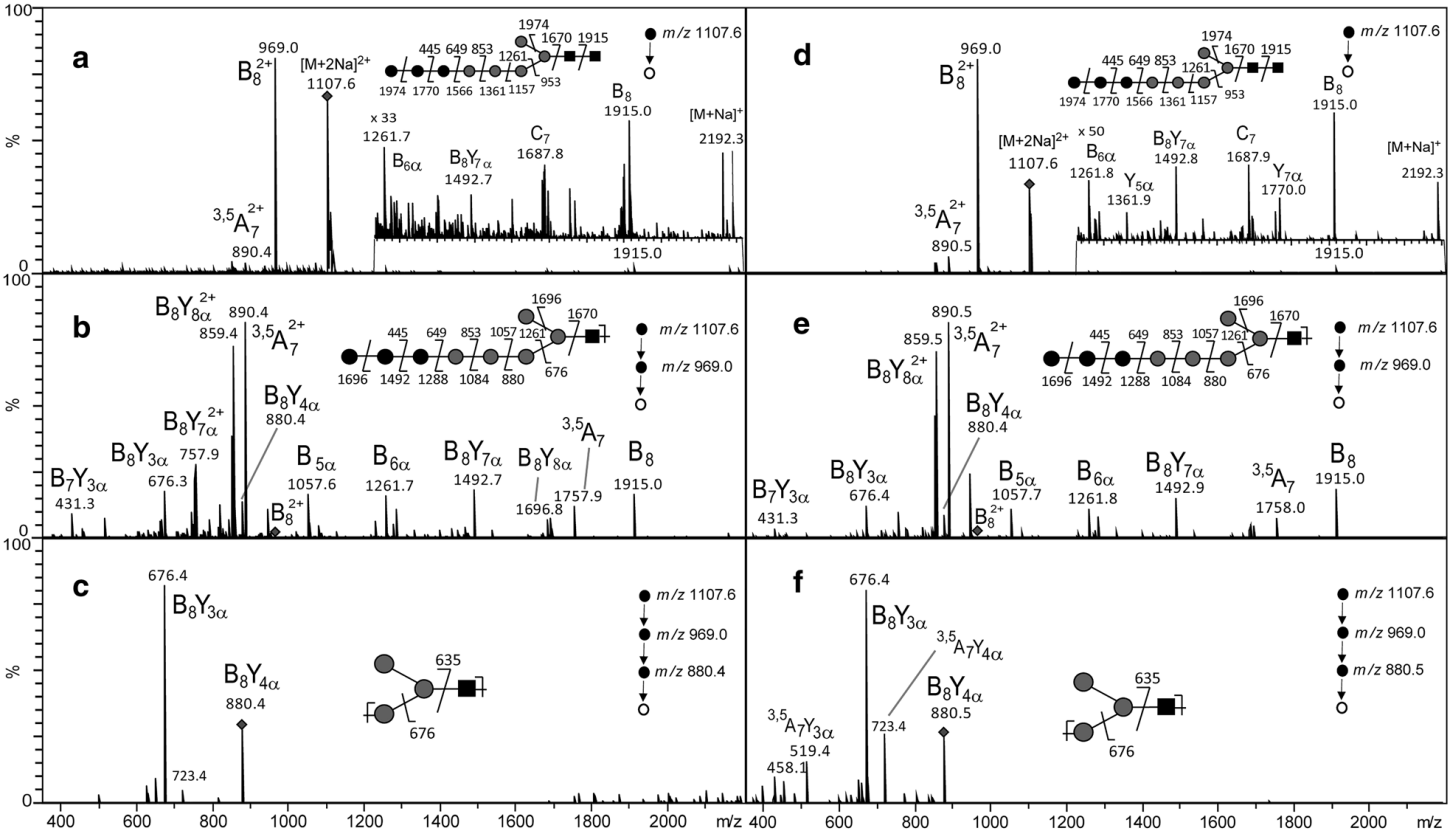
Multistage tandem mass spectrometry analysis of the permethylated LLO-released oligosaccharide isolated from *C. reinhardtii XTA* mutant (**a**–**c**) and from wild-type (**d**–**f**). ESI-MS^n^ spectra with n = 2 (**a** and **d**), n = 3 (**b** and **e** and n = 4 (**c** and **f**) of the [M + 2Na]^2+^ *m/z* 1107.6 precursor ion of the permethylated Hex_8_HexNAc_2_ derivative isolated from either the *C. reinhardtii XTA* mutant or the wild-type cells. The precursor ion selected for the fragmentation analysis is shown with a diamond and its fragmentation pattern is proposed according to Prien et al. [40]. Black square: *N*-acetylglucosamine; grey circle: mannose, black circle: glucose. The fragment ions are labelled according to the nomenclature of Domon and Costello [41]

**Fig. 3:**
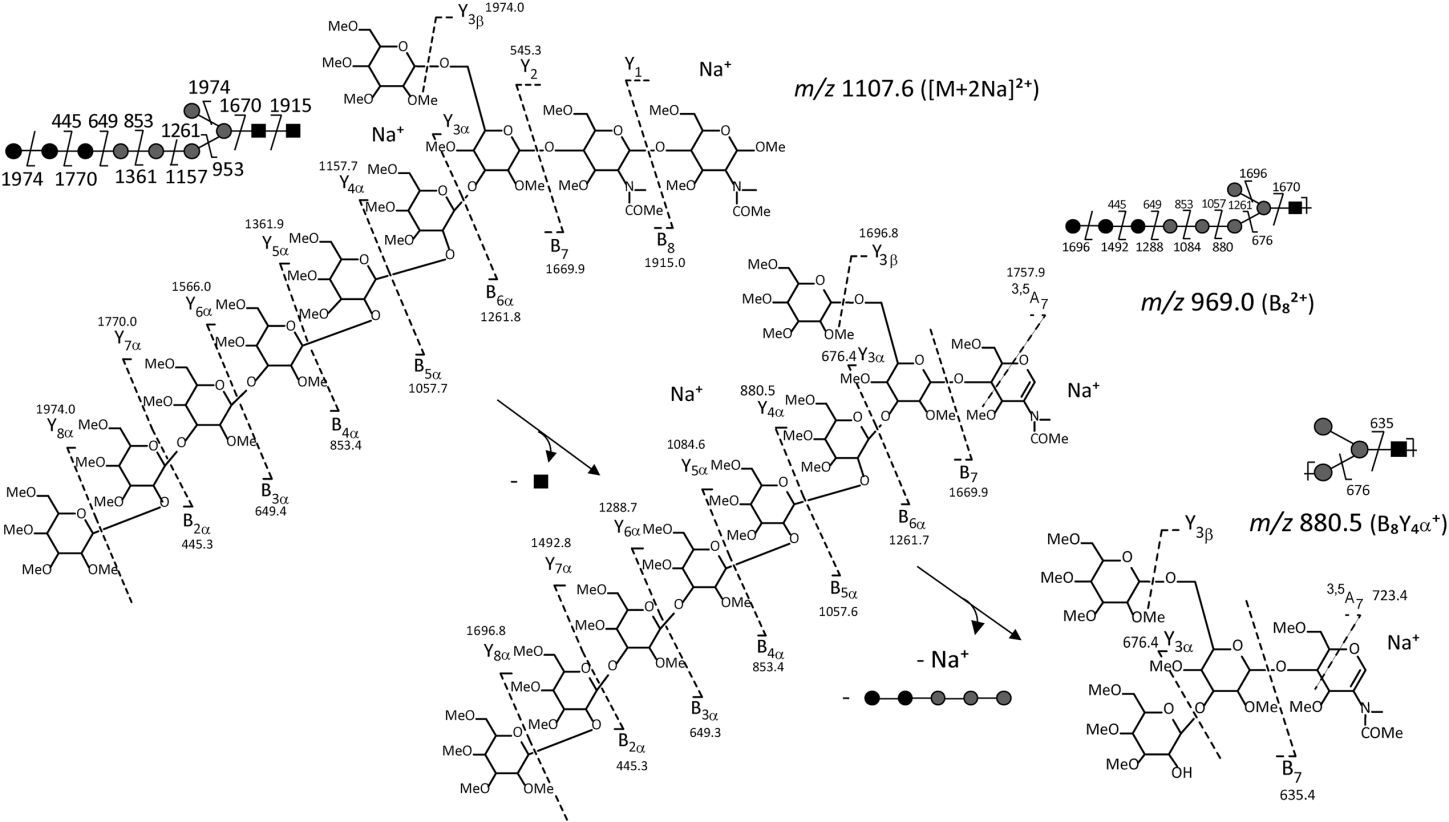
Scheme representing the fragmentation pattern for *m/z* 1107.6 precursor ion ([M + 2Na]^2+^) of permethylated Hex_8_HexNAc_2_ derivative isolated from the LLO of the *C. reinhardtii XTA* mutant or the wild-type cells. Cleavages of the glycosylic bond and cross ring cleavages are represented by dotted lines. The fragment ions are labelled according to the nomenclature of Domon and Costello [41]

The optimised protocol was first applied to the characterization of the LLO-released oligosaccharide isolated from the LLO of two *C. reinhardtii* mutants which are impaired in two different genes encoding putative xylosyltransferases (*XTA* and *XTB*) involved in the xylosylation of complex-type *N*-glycans in *C. reinhardtii* [18, 38, 39]. In parallel, wild-type *C. reinhardtii* cells were submitted to the same LLO extraction protocol. After extraction, mild hydrolysis and permethylation, LLO-released oligosaccharides of *XTA, XTB* mutants and wild-type have been analyzed using MALDI-TOF–MS. The resulting mass spectra for all three samples show a predominant ion at *m/z* 2192 which was assigned to be the sodium adduct of a Hex_8_HexNAc_2_ (data not shown). To gain insight into the structural information about this LLO-released oligosaccharide, multistage tandem mass spectrometry (ESI-MS^n^) experiments have been performed, as this method has been proven to be powerful to determine the chemical structures of polysaccharide fragments [33, 34, 37] and glycans [23, 40]. Here, the [M + 2Na]^2+^ ion (*m/z* 1107.6) of Hex_8_HexNAc_2_ has been selected for fragmentation (Fig. 2). The ESI-MS^*n*^ (*n* = 2, 3 and 4) spectra show mainly B- and Y-ion series which are fragment ions usually observed in MS/MS analyses of oligosaccharides [23, 37, 41]. They arise from cleavages of the glycosidic bond with loss of either the reducing terminal portion for B-ions or the non-reducing terminal portion for Y-ions. Few BY- ions resulting from two glycosidic cleavages as well as some A-ions arising from cross ring cleavages were also observed. The fragment ions are labelled according to the nomenclature introduced by Domon and Costello in 1988 [41]. For clarity in the figures, we did not detail all the fragment ions observed on the MS^n^ spectra, but we choose to focus on those corresponding to diagnostic transitions which allow determining the oligosaccharide structure and even discriminating potential isomers. Thus, the specific fragmentation pathway *m/z* 1107.6 ([M + 2Na]^2+^) →  *m/z* 969.0 (${\text{B}}_{8}^{2 + }$) → *m/z* 880.4 (B_8_Y_4α_) → *m/z* 676.4 (B_8_Y_3α_) which was observed in both mutants (Fig. 2a–c for *C. reinhardtii XTA* mutant and Additional file 1 for *C. reinhardtii XTB* mutant) as well as in wild-type strains (Fig. 2d–f) is consistent with the oligosaccharide structure previously described for the LLO of the *C. reinhardtii* wild-type cells [23]. Figure 3 shows the fragmentation pattern of the [M + 2Na]^2+^ ion (*m/z* 1107.6) with assignments and detailed structures of the diagnostic fragment ions. These results confirmed the presence of a linear LLO structure in *C. reinhardtii XTA* and *XTB* mutants as compared to the wild-type cells. Indeed, as in wild-type, *C. reinhardtii XTA* and *XTB* mutants accumulate a linear LLO-released oligosaccharide containing 8 hexose and 2 *N*-acetylglucosamine residues which largely differs from the Glc_3_Man_9_GlcNAc_2_ synthetized in most of the eukaryotes [9, 11]. The bioinformatic analysis of *C. reinhardtii* genome failed in predicting orthologous genes for ALG3, 9 and 10 responsible for the completion of the LLO synthesis into Glc_3_Man_9_GlcNAc_2_-PP-Dolichol [18, 22, 38]. Based on these predictions, *C. reinhardtii* LLO-released oligosaccharide structure should be a linear Glc_2_Man_5_GlcNAc_2_. However, the MS analysis suggested the accumulation in *C. reinhardtii* of a Hex_8_HexNAc_2_ LLO–released oligosaccharide, thus exhibiting one additional hexose residue than the LLO structure predicted from genomic data. Therefore, a non-yet identified glycosyltransferase should act on the LLO precursor to transfer this additional hexose. As the specific ion transitions *m/z* 1107.6 → *m/z* 969.0 → *m/z* 880.4 → *m/z* 676.4 evidenced by ESI-MS^n^ analyses is consistent with the linear glycan structure depicted in Figs. 2 and 3, we proposed that LLO from *C. reinhardtii* mutants exhibit a trihexyl extension which could probably be a triglucosyl one. Glc_3_Man_5_GlcNAc_2_ LLO-released oligosaccharide may result from the action of an additional glucosyltransferase adding a third glucose to the LLO precursor. This hypothesis is further supported by the prediction in the *C. reinhardtii* genome of a putative alpha (1, 2) glucosidase I (GSI) [18, 22, 38]. This enzyme would be responsible for the cleavage of the terminal glucose of the triglucosyl extension on the glycan precursor after its transfer onto the nascent glycoprotein [42, 43].

### Identification of the LLO-released oligosaccharide from *Phaeodactylum tricornutum*

**Fig. 4:**
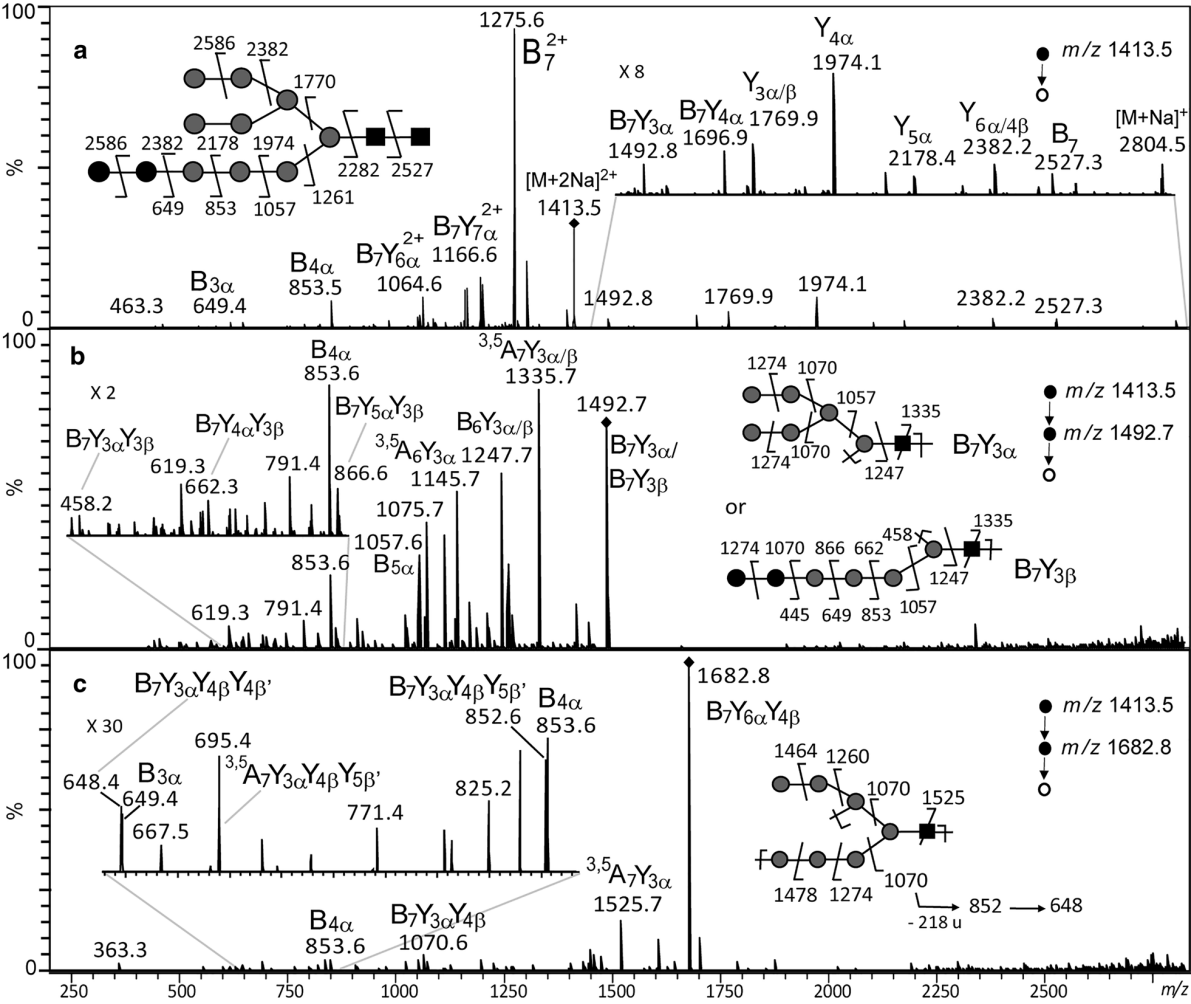
Multistage tandem mass spectrometry analysis allowing the determination of the structure of the permethylated LLO-released oligosaccharide isolated from the diatom *P. tricornutum*. ESI-MS^*n*^ spectra with *n* = 2 selecting *m/z* 1413.5 ([M + 2Na]^2+^) as the precursor ion (**a**), *n* = 3 selecting *m/z* 1492.5 ([M + Na]^+^) as intermediate ion (**b**) and *n* = 3 with *m/z* 1682.5 ([M + Na]^+^) as intermediate ion (**c**) of permethylated Hex_11_HexNAc_2_ derivative) isolated from *P. tricornutum.* On each panel, the ion selected for the fragmentation analysis is shown with a diamond and its fragmentation pattern is proposed according to Prien et al. [40]. Black square: *N*-acetylglucosamine; grey circle: mannose, black circle: glucose. The fragment ions are labelled according to the nomenclature of Domon and Costello [41]

**Fig. 5:**
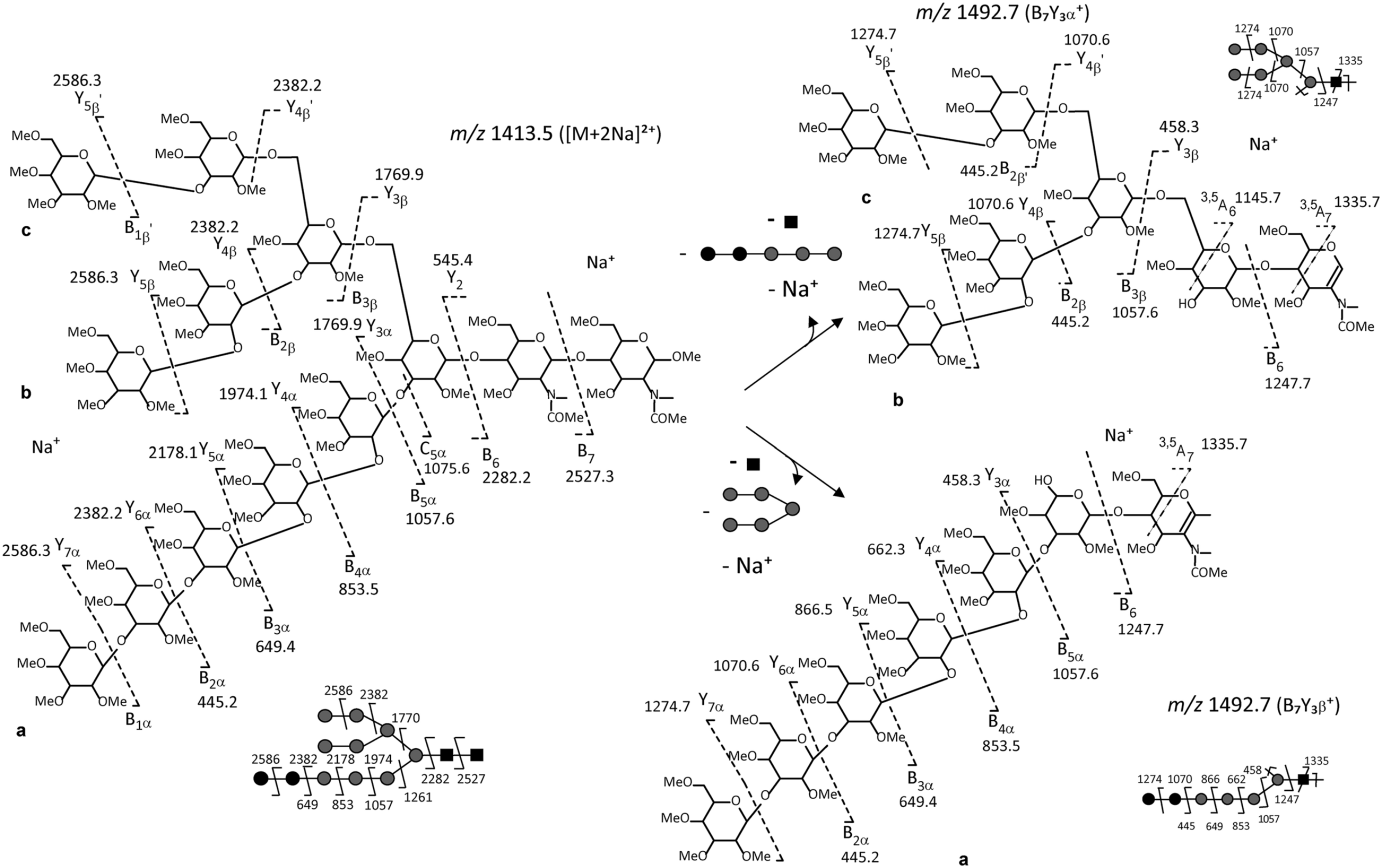
Scheme representing the fragmentation pattern for *m/z* 1413.5 precursor ion ([M + 2Na]^2+^) and *m/z* 1492.7 intermediate ion of permethylated Hex_11_HexNAc_2_ isolated from the LLO of *P. tricornutum.* Cleavages of the glycosylic bond and cross ring cleavages are represented by dotted lines. The fragment ions are labelled according to the nomenclature of Domon and Costello [41]

**Fig. 6:**
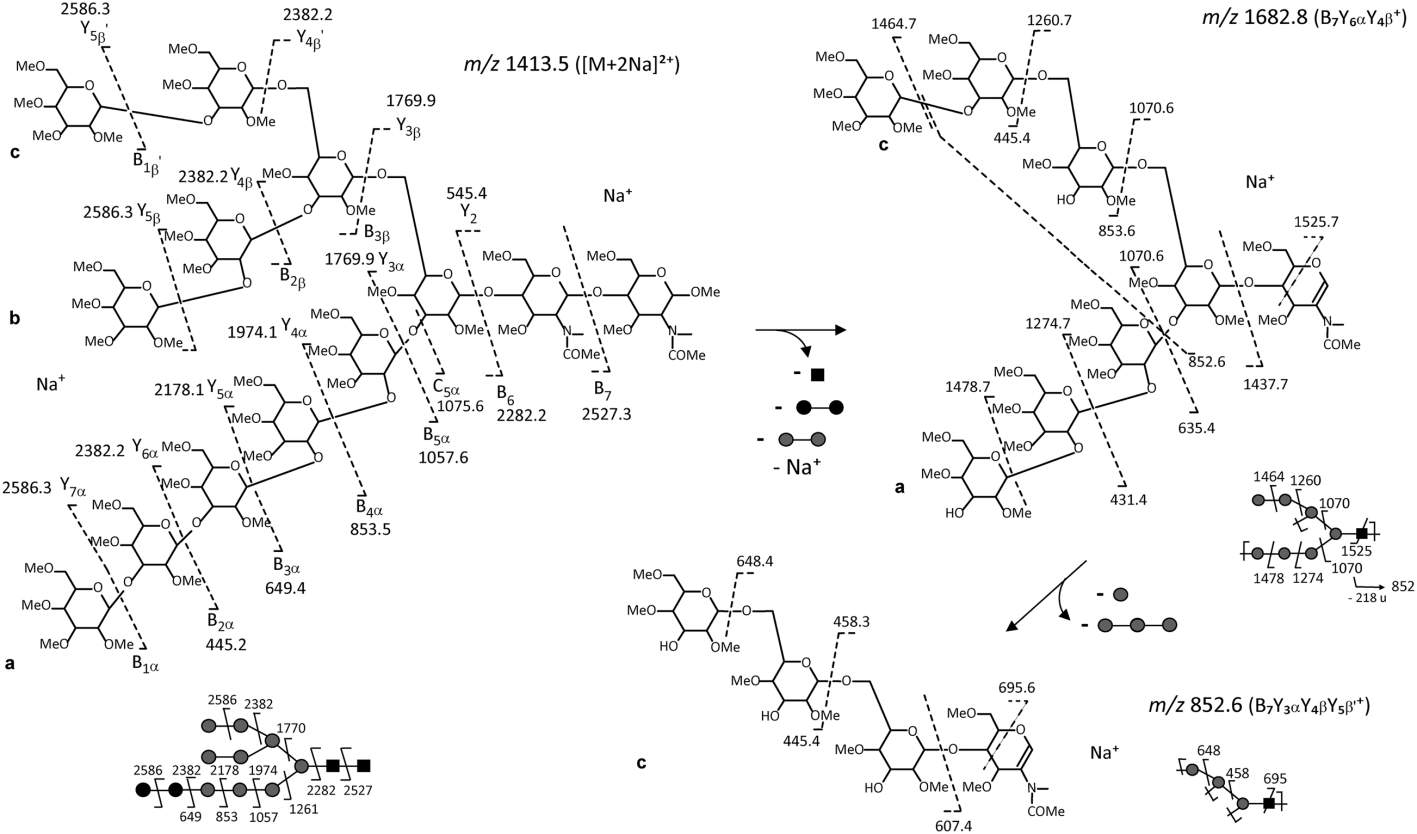
Scheme representing the fragmentation pattern for *m/z* 1413.5 precursor ion ([M + 2Na]^2+^) and *m/z* 1682.8 intermediate ion of permethylated Hex_11_HexNAc_2_ isolated from the LLO of *P. tricornutum.* Cleavages of the glycosylic bond and cross ring cleavages are represented by dotted lines. The fragment ions are labelled according to the nomenclature of Domon and Costello [41]

To investigate whether the developed method would be suitable for other microalgae species, we investigated the oligosaccharide structure released from the LLO of the diatom *P. tricornutum* which has never been reported so far in the literature. LLO was isolated using the same protocol and its oligosaccharide moiety was cleaved from the dolichol lipid anchor and permethylated prior to MS analysis. MALDI-TOF mass spectrum exhibited two ions at *m/z* 2804 and *m/z* 2820 (data not shown). They respectively correspond to sodium and potassium adducts of a Hex_11_HexNAc_2_ structure, differing by one hexose residue from the Glc_3_Man_9_GlcNAc_2_ LLO precursor identified in mammals and land plants [9, 11, 44]. In addition, ESI-MS^n^ (n = 1 to 4) analyses have been performed by selecting the [M + 2Na]^2+^ of Hex_11_HexNAc_2_ structure as a precursor ion (*m/z* 1413.5). ESI-MS^*n*^ (*n* = 3 and 4) experiments (Fig. 4 and Additional files 2, 3 and 4) were performed and were consistent with the tri-antennae structure proposed in Figs. 4a, 5, 6 and Additional file 2. For example, the ESI-MS^3^ spectrum selecting *m/z* 1492.7 as the intermediate fragment ion (Figs. 4b, 5) showed the presence of the discriminant fragment ions *m/z* 458.2 (B_7_Y_3α_Y_3β_) *m/z* 662.3 (B_7_Y_4α_Y_3β_) and *m/z* 866.6 (B_7_Y_5α_Y_3β_) of the antennae a in addition to *m/z* 1070.6 (B_7_Y_6α_Y_3β_) and *m/z* 1274.7 (B_7_Y_7α_Y_3β_) which are not discriminant, because the two latter can arise from the fragmentation of either the antenna **a** or the di-antenna branch **bc**. It is worth noting that *m/z* 1492.7 first fragments into *m/z* 1274.7 by elimination of 218 u (terminal hexose residue). Then, successive losses of 204 u (hexose residue that was linked with two other residues) explain the ion series *m/z* 1070.6, *m/z* 866.6, *m/z* 662.3 and *m/z* 458.2. This fragmentation pattern is characteristic of the antennae a (Fig. 5). The ESI-MS^3^ spectrum selecting *m/z* 1682.5 as the intermediate fragment ion (Figs. 4c, 6) revealed the presence of discriminant fragment ions at *m/z* 852.6 and *m/z* 648.4 corresponding respectively to the Hex_3_HexNAc and Hex_2_HexNAc structures arising from the fragmentation of a branched LLO. It is worth noting that *m/z* 1682.5 can also be assigned to the structure depicted in the Additional file 2 that gives rise to the fragment ions *m/z* 649.3 (B_3α_) and *m/z* 853.6 (B_4α_) in Fig. 4c. The presence of a tri-antenna LLO-released oligosaccharide structure was also evidenced by the ESI-MS^4^ spectrum showing the fragmentation pattern “*m/z* 1 413.5 → *m/z* 1 057.6 → *m/z* 839.4 → product ions (Additional file 3). The fragment ions *m/z* 417.2 and *m/z* 621.3 can only result from fragmentation of a branched ion *m/z* 839.4 (B_3β_Y_5β_ from antenna bc) while *m/z* 431.2 can only arise from fragmentation of a linear *m/z* 839.4 (B_5α_Y_7α_) characteristic of antenna a. Moreover, the presence of tri-antenna was also confirmed by complementary ESI-MS^n^ experiments (Additional file 4). From these data, two structures, Glc_3_Man_8_GlcNAc_2_ or Glc_2_Man_9_GlcNAc_2_, can be proposed. The genomic analysis favors the Glc_2_Man_9_GlcNAc_2_ for the structure of the oligosaccharide released from the LLO. Indeed, bioinformatics analysis of *P. tricornutum* genome reveals the presence of all the enzymes involved in the synthesis of the precursor, except ALG10 and the GSI which are respectively involved in the addition and removal of the third glucose residue from the LLO precursor [16, 45]. Moreover, previous analysis of the *N*-glycans attached to the endogenous proteins of *P. tricornutum* showed a high abundance of oligomannosidic *N*-glycans especially the one composed of 9 mannose residues [16]. Thus, all together, these results demonstrate that the structure of the oligosaccharide released from the LLO of *P. tricornutum* is a Glc_2_Man_9_GlcNAc_2_ structure.

## Conclusions

In this communication, we described a simple and user-friendly protocol to extract and analyze the oligosaccharide structure of the LLO from microalgae. This method allows to collect enough material to perform MS detailed analyses, thus representing a powerful approach allowing identification of the structure of the LLO-released oligosaccharide. Moreover, the contribution of multistage tandem mass spectrometry to elucidate chemical structure of complex polymers is once more comforted. The advantage of this technique over radio-labelling methods is the access to the structural identification in close details of either known structures or those to elucidate. The validity of the method has been confirmed by comparison of wild-type and mutant data from *C. reinhardtii* and the elucidation of the LLO-released oligosaccharide structure of *P. tricornutum*. These results illustrate the adaptability of the optimized method as it allows successful identification of the LLO-released oligosaccharide of the diatom, *P. tricornutum* as well as the one from *C. reinhardtii* which belongs to the chlorophyceae lineage. Now, it could be conceived to use this user-friendly method to extract and analyse the LLO-released oligosaccharide structures in other microalgae, allowing understanding of the evolution of the LLO.

## Methods

The water used throughout the protocol was deionized water. LC–MS grade water was purchased from Thermo-Scientific and was used for the purification of the LLO-released oligosaccharide. All the extraction steps up to the mild acidic hydrolysis were performed at 4 °C.

### *Chlamydomonas reinhardtii* cell culture and lysis

The *C. reinhardtii* mutant strains *XTA* (LMJ.RY0402.087519) and *XTB* (LMJ.RY0402.118417) were ordered from the CLIP library (https://www.chlamylibrary.org). Those mutants were generated by random insertion of a paromomycin resistance cassette (CIB1) [46]. Cassette occupancy site in those two mutants had been checked as recommended in [46].

One liter of *C. reinhardtii* cell culture was grown in TAP medium (Tris Acetate Phosphate) at 23 °C, agitated at 150 rpm until reaching an optical density (OD) at 680 nm of 1. A centrifugation at 4500 × *g* using a Beckman Coulter Allegra^®^ X-15R centrifuge with SX4750A rotor for 10 min was then performed in order to pellet the cells. The cells were washed twice with 20 mM phosphate buffer pH 7.4. Then, the cell pellets were resuspended in 6 mL of 20 mM phosphate buffer pH 7.4 containing 1 mM ethylene diamine tetraacetic acid, 0.5 mM dithiothreitol and 0.5 mM phenylmethylsulfonyl fluoride (PMSF). This solution was prepared extemporaneously. Cells were grinded using glass beads in the Lysing Matrix D tubes (MPbiomedicals) and a homogenizer (Fast Prep-24 5G™ High Speed Homogenizer, MPbiomedicals). Five cycles of 30 s at 6.5 m s^−1^ with chilling for 2 min on ice between each cycle were performed. Samples were then centrifuged at 10,000 × *g* for 10 min at 4 °C and the supernatant was transferred in ultracentrifugation tubes (Beckman Coulter). Each remaining pellet was resuspended in 1 mL of the same extraction buffer and then, 5 new cycles of 30 s at 6.5 m s^−1^ with chilling for 2 min on ice between cycles were achieved. The resulting supernatants containing the cell lysate (S1) were pooled with the previous ones.

### *Phaeodactylum tricornutum* cell culture and lysis

One liter of Pt 1.8.6 strain (CCAP 1055/1) was grown in sea water (33 g L^−1^ of Instant Ocean Sel Marin, Truffaut) enriched with 0.1% (v/v) of Conway medium and 0.2% (v/v) of 40 g L^−1^ sodium silicate [16] until reaching 1.10^7^ cells mL^−1^. The culture medium was then centrifuged at 4500 × *g* using Beckman Coulter Allegra^®^ X-15R (with SX4750A rotor) for 10 min to pellet the cells. The resulting cell pellets were resuspended as described for *C. reinhardtii* cells. The diatom cells were grinded using a homogenizer (Fast Prep-24 5G™ High Speed Homogenizer, MPbiomedicals) and the Lysing Matrix E tubes (MPbiomedicals) performing 6 cycles of 30 s at 6,5 m s^−1^ with chilling for 2 min on ice between cycles. Samples were then treated as per *C. reinhardtii*. The resulting supernatants contained the diatom cell lysate were named S1.

### Microsomal fraction preparation

S1 was centrifuged at 20,000×*g* at 4 °C in a Beckman Coulter ultracentrifuge Optima™ XL-100 K for 30 min using a type 70.1Ti rotor. The resulting pellet was discarded and the supernatant (S2) was centrifuged at 100,000×*g* in the same conditions for 1 h. The new supernatant (S3) was discarded and the resulting pellet containing the microsomal fraction (MP) was conserved for further LLO purification steps.

### Solvent extraction

The microsomal fraction was resuspended with 400 μL of water (LC–MS Grade, Fisher Chemical) in a glass tube, 800 μL of methanol (LC–MS Grade, Fisher Chemical) and 1.2 mL of chloroform (ACS reagent grade, Acros Organics) were added (1/2/3, v/v/v). The sample was then homogenized by vortex and centrifuged at 4000×*g* at 4 °C for 2 min in the Beckman Coulter Allegra^®^ X-15R centrifuge using the SX4750A rotor. After centrifugation, the sample exhibited a soluble upper phase (UP), an insoluble lower phase (LOW) separated by an interphase (INTER) (Fig. 1). The lower phase (LOW) containing pigments, was gently discarded using a glass pipette.

Three mL of the mixture A (Mix A in Fig. 1) containing chloroform/methanol and 4 mM MgCl_2_ in ultrapure water (86/14/1, v/v/v) was then added to the remaining upper phase. After mixing and centrifugation at 4000×*g* for 2 min at 4 °C, the lower phase was discarded. This step was repeated 3 times to remove all pigments. The remaining upper phase (UP, Fig. 1) was then washed four times with 3 mL of the mixture B (Mix B in Fig. 1) containing methanol/4 mM MgCl_2_ in water/chloroform (16/15/1, v/v/v). After homogenizing by vortex and centrifuging (4000×*g* at 4 °C for 2 min), the supernatant was discarded. This step was performed 3 times to remove soluble proteins. Finally, the pellet partially air dried, was re-suspended three times with 3 mL of the mixture C (Mix C in Fig. 1) containing chloroform/methanol/water (10/10/3, v/v/v). After homogenization by vortex and centrifugation (4000×*g* at 4 °C for 2 min), the supernatant containing the LLO was transferred in a new glass tube and dried with air stream.

### Mild acid treatment

In order to remove the dolichol lipid moiety from the oligosaccharide, dried LLO was hydrolyzed by a mild acid treatment using 0.1 M of TFA (LC–MS grade, Thermo Scientific) at 80 °C for 1 h. In these conditions, the glycosidic bond between the dolichol phosphate and the oligosaccharide was efficiently hydrolyzed. Released oligosaccharides, later called LLO-released oligosaccharide, were then lyophilized.

### LLO oligosaccharides purification

LLO-released oligosaccharides were resuspended in 500 µL of water (LC–MS grade, Fisher Chemical) and then purified using carbograph columns (Hypersep™ Hypercarb™ SPE Cartridges, 200 mg, 3 mL, ThermoFisher Scientific). First, the columns were conditioned by successive washing with 3 mL of 1 M sodium hydroxide (Laboratory reagent grade, Fisher scientific), 6 mL of water (LC–MS grade, Fisher Chemical), 3 mL of acetic acid 30% (v/v) Acros Organics) and finally 3 mL of water (LC–MS grade, Fisher Chemical). These steps may be carried out under vacuum (5 mmHg) using a Manifold. Then, the columns were successively washed with 3 mL of 50% acetonitrile in water (v/v) (LC–MS grade, Fisher Chemical), 6 mL of 5% acetonitrile in water (v/v) and finally with 6 mL of water (LC–MS grade, Fisher Chemical). The LLO-released oligosaccharide samples were resuspended in 500 µL of water (LC–MS grade, Fisher Chemical) and were loaded into the column. After washing with 3 mL of water and 3 mL of 5% acetonitrile in water (v/v), LLO-released oligosaccharides were eluted by 4 × 0.5 mL of 50% acetonitrile in 0.1 M of TFA (v/v). The eluted fractions were recovered and were lyophilized in glass tubes prior to permethylation.

### Permethylation

A suspension of sodium hydroxide in dimethylsulfoxyde was prepared by grinding about 0.90 g of dried sodium hydroxide (4 tablets) in 3 mL of dimethylsulfoxyde (Sigma-Aldrich). 0.6 mL of this preparation was added to the lyophilized sample. Then, 0.5 mL of iodomethane (ACS reagent grade, Acros Organics) was added and the sample was incubated under agitation for 1 h. One mL of water was added to quench the reaction. One mL of chloroform and 3 mL of water (LC–MS grade, Thermo Scientific) were then added to the samples and mixed. When two phases appeared clearly, the upper aqueous phase was discarded, and 3 mL of water were added. This step was repeated until pH was neutralized. The chloroform phase containing permethylated glycans was then dried.

### Permethylated LLO oligosaccharides purification

After permethylation, the LLO-released oligosaccharide was purified using C18 columns (Hypersep™ C18 Cartridges, 200 mg, 3 mL, Thermo Fisher). Columns were conditioned by successive washing with 5 mL of methanol, 5 mL of water (LC–MS grade, Fisher Chemical), 5 mL of acetonitrile and finally with 5 mL of water (LC–MS grade, Fisher Chemical). Permethylated sample was resuspended in 200 μL of 80% methanol (v/v) and were loaded on the column. Successive elutions with 2 mL of 15% (v/v), 35% (v/v) and 75% acetonitrile (v/v) were performed. Permethylated oligosaccharides were recovered in the 75% acetonitrile fraction. The resulting samples were then dried in a Thermofisher SPD111 V SpeedVac^®^. After drying, the samples were ready to be analyzed using the Matrix Assisted Laser Desorption Ionization—Time Of Flight Mass Spectrometry and ElectroSpray Ionization Multistage tandem Mass Spectrometry (ESI-MS^n^).

### Matrix assisted laser desorption ionization—mass spectrometry (MALDI-TOF–MS)

MALDI-TOF–MS experiments were performed using an Autoflex III time-of-flight mass spectrometer (Bruker Daltonics (Bremen, Germany) equipped with a Nd: YAG laser (355-nm wavelength), FlexControl 3.3 and FlexAnalysis 3.3 software package. Spectra were acquired in the positive-ion reflector mode using a mass range *m/z* 500–4000, 100 Hz laser frequency, 10 ns pulsed ion extraction delay and 50% attenuation laser. At least 3 000 individual spectra were averaged. The instrument accelerating voltage was 19 kV. External calibration of MALDI-TOF mass spectra was carried out using singly charged monoisotopic peaks of angiotensin II (5 pmol μL^−1^), substance P (5 pmol μL^−1^), ACTH 18–39 fragment (5 pmol μL^−1^) and insulin B (10 pmol µL^−1^) in the same experimental (matrix conditions, sample deposit,…) and instrumental conditions (accelerating voltage, laser frequency, ion extraction delay, attenuation laser,…) than those used for the LLO-released oligosaccharide sample analyses. 2,5-dihydrohybenzoic acid (DHB) was used as matrix: 1 µL of sample was mixed within 1 µL of matrix solution (10 mg mL^−1^ in 0.1% aqueous TFA/acetonitrile (70/30: v/v)), then 0.7 µL of the sample/matrix mixture was spotted and dried onto the MALDI polished stainless-steel target plate (Bruker Daltonics, Bremen, Germany).

### Electrospray ionization multistage tandem mass spectrometry (ESI-MS^n^)

ESI-MS^*n*^ (*n* = 1 to 4) experiments were performed using a Bruker HCT Ultra ETD II quadrupole ion trap (QIT) mass spectrometer equipped with an electrospray source, the Esquire control 6.2 and Data Analysis 4.0 softwares (Bruker Daltonics, Bremen, Germany). The ESI parameters were as followed: capillary and end plate voltages respectively set at − 4.0 kV and − 3.5 kV in positive ion mode, skimmer and capillary exit voltages set at 40 V and 120 V, respectively, nebulizer gas (N_2_), pressure, drying gas (N_2_) flow rate and drying gas temperature were 10 psi, 7.0 L min^−1^ and 300 °C, respectively. The data were acquired using a mass range of either *m/z* 200–2200 range (for Hex_8_HexNAc_2_) or *m/z* 200–2850 range (for Hex_11_HexNAc_2_) and using a scan speed of *m/z* 8100 per second. The number of ions entering the trap cell was automatically adjusted by controlling the accumulation time using the ion charge control (ICC) mode (target 100,000) with a maximum accumulation time of 50 ms. The injection low-mass cut-off (LMCO) value was *m/z* 90 (for Hex_8_HexNAc_2_) or *m/z* 140 for Hex_11_HexNAc_2_ derivatives. The values of spectra average and rolling average were 6 and 2, respectively. ESI-MS^*n*^ experiments were carried out by collision-induced dissociation (CID) using helium as the collision gas, isolation width of 1 or 1.5 *m/z* unit for the precursor ions and for the intermediate ions using a resonant excitation frequency with an amplitude from 0.8 to 1.0 Vp–p. Sample solutions (resuspension in 50 µL of CH_3_CN/H_2_O were infused into the source at a flow rate of 180 µL h^−1^ by means of a syringe pump (Cole-Palmer, Vernon Hills, IL, USA). External calibration was performed using the ‘tuning mix’ from Agilent Technologies (Santa Rosa, CA, USA). All mass spectra were manually interpreted, based on literature [23, 40, 41]. The Glycoworkbench software v2.1 was used to support and confirm the assignment of the fragment ions. The ESI-MS^*n*^ spectra showed diagnostic fragment ions resulting from the characteristic fragmentation pathways of a specific chemical structure and consequently helped in identifying the oligosaccharide structure according to Prien et al. [40]. The extraction of LLO and MS analyses have been performed at least twice independently.

## Additional files

**Additional file 1.** Multistage tandem mass spectrometry analyses of the structure of the LLO-released oligosaccharide isolated from C. reinhardtii XTB mutant by ESI-MS^n.^ ESI-MS^n^ spectra with *n* = 2 (panel A), *n* = 3 (panel B) and *n* = 3 (panel C) of permethylated Hex_8_HexNAc_2_ derivative (*m/z* 1107.6 corresponding to [M + 2Na]^2+^ precursor ion) isolated from *C. reinhardtii XTB* mutant. On each panel, the ion selected for the fragmentation analysis is shown with a diamond and its fragmentation pattern is proposed according to Prien et al. [40]. Black square: *N*-acetylglucosamine; grey circle: mannose, black circle: glucose. The fragment ions are labelled according to the nomenclature of Domon and Costello [41].

**Additional file 2.** Scheme representing a fragmentation pathway complementary to the one depicted in Fig. 5. Cleavages of the glycosylic bond and cross ring cleavages are represented by dotted lines. The fragment ions are labelled according to the nomenclature of Domon and Costello [41].

**Additional file 3.** Multistage tandem mass spectrometry analyses of the structure of the LLO-released oligosaccharide isolated from *P. tricornutum*. ESI-MS^n^ spectra with *n* = 3 (panel A) and *n* = 4 (panel B) of permethylated Hex_11_HexNAc_2_ derivative (*m/z* 1413.5 corresponding to [M + 2Na]^2+^ precursor ion) isolated from *P. tricornutum.* On each panel, the ion selected for the fragmentation analysis is shown with a diamond and its fragmentation pattern is proposed according to Prien et al. [40]. Black square: *N*-acetylglucosamine; grey circle: mannose, black circle: glucose. The fragment ions are labelled according to the nomenclature of Domon and Costello [41]. Note that loss of 204 u correspond to the elimination of a 1,2-; 1,3-; 1,4- or 1,6-linked hexose residue while loss of 218 u correspond to the elimination of a terminal hexose residue. Therefore, the successive losses of two 218 u (*m/z* 1057.5 → *m/z* 839.4 → *m/z* 621.3) indicate a di-antenna ion *m/z* 1057.5 while the successive losses of 204 u (*m/z* 1057.5 → *m/z* 853.4 → *m/z* 649.3) indicate a linear ion *m/z* 1057.5.

**Additional file 4.** Multistage tandem mass spectrometry analyses of the structure of the LLO-released oligosaccharide isolated from *P. tricornutum*. ESI-MS^n^ spectra with *n* = 3 (panels A, C and E) and *n* = 4 (panel B and D) of permethylated Hex_11_HexNAc_2_ derivative (*m/z* 1413.5 corresponding to [M + 2Na]^2+^ precursor ion) isolated from *P. tricornutum.* On each panel, the ion selected for the fragmentation analysis is shown with a diamond and its fragmentation pattern is proposed according to Prien et al. [40]. Black square: *N*-acetylglucosamine; grey circle: mannose, black circle: glucose. The fragment ions are labelled according to the nomenclature of Domon and Costello [41]. Note that loss of 190 u correspond to the elimination of a hexose residue which was linked with three other residues in the oligosaccharide (for example, a 1,3,6-linked hexose residue). Therefore, the successive losses of two 218 u (*m/z* 1247.6 → *m/z* 1029.5 → *m/z* 811.5) indicate a di-antenna ion *m/z* 1247.6 while the loss of 190 u followed by successive losses of 204 u (*m/z* 1247.6 → *m/z* 1057.5 → *m/z* 853.5 → *m/z* 649.4 → *m/z* 445.3) indicate a linear ion *m/z* 1247.6.

## Abbreviations

ALG: asparagine-linked glycosylation
ESI: electrospray ionization
Glc: glucose
GlcNAc: *N*-acetylglucosamine
GSI: alpha glucosidase I
Hex: hexose
LLO: lipid-linked oligosaccharide
Man: mannose
MALDI-TOF: matrix assisted laser desorption ionization—time of flight
MS: mass spectrometry
PMSF: phenylmethylsulfonyl fluoride
PP-Dol: dolichol pyrophosphate
XTA: xylosyltransferase mutant A
XTB: xylosyltransferase mutant B
