# Supplementary figures and images for: User-friendly extraction and multistage tandem mass spectrometry based analysis of lipid-linked oligosaccharides in microalgae
Source: Plant Methods. 2018 Dec 5;14:107. doi: 10.1186/s13007-018-0374-8 (PMC6280548; doi:10.1186/s13007-018-0374-8)

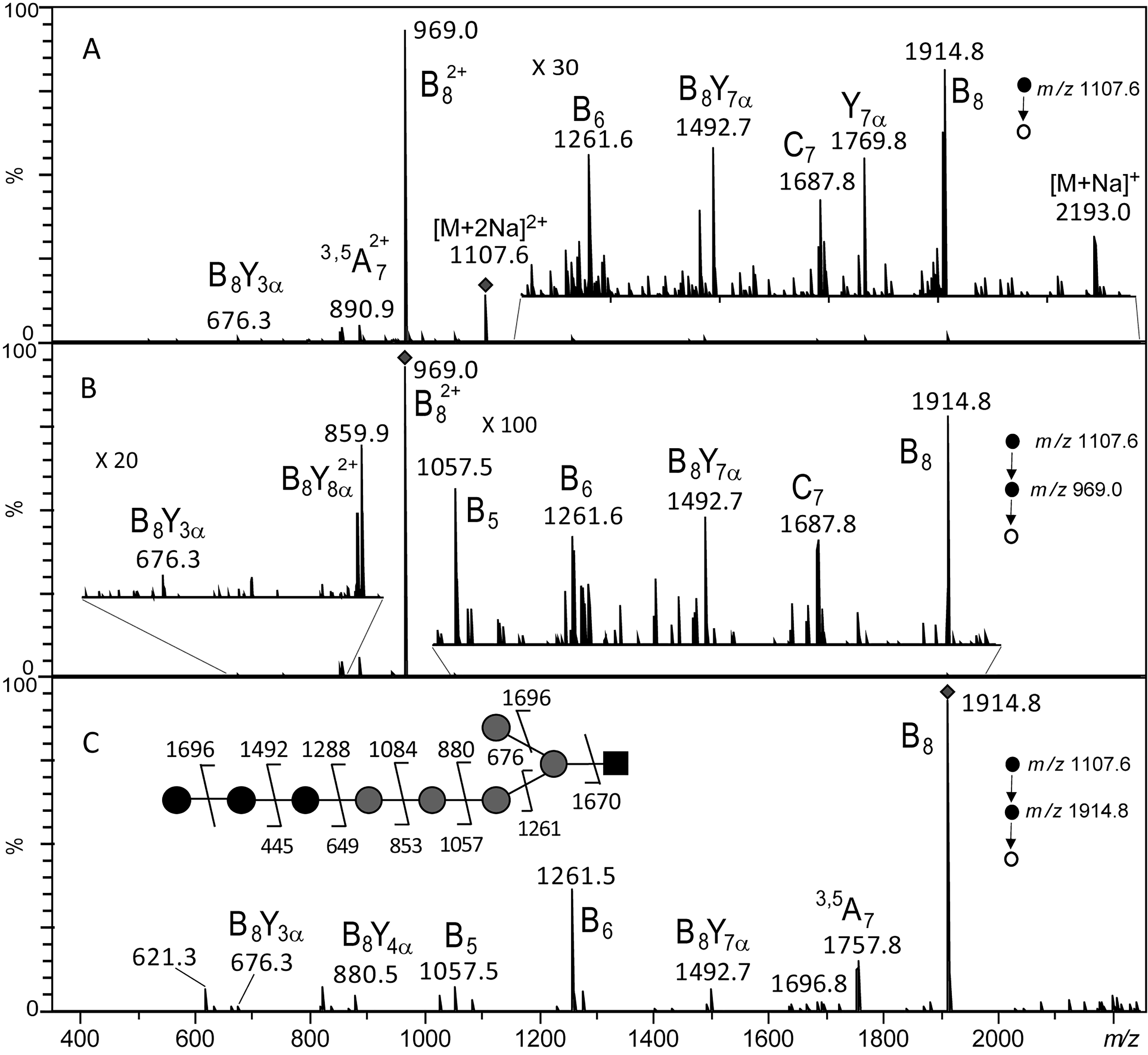

Supplement: Supplementary file 1 — Additional file 1. Multistage tandem mass spectrometry analyses of the structure of the LLO-released oligosaccharide isolated from C. reinhardtii XTB mutant by ESI-MSn. ESI-MSn spectra with n = 2 (panel A), n = 3 (panel B) and n = 3 (panel C) of permethylated Hex8HexNAc2 derivative (m/z 1107.6 corresponding to [M + 2Na]2+ precursor ion) isolated from C. reinhardtii XTB mutant. On each panel, the ion selected for the fragmentation analysis is shown with a diamond and its fragmentation pattern is proposed according to Prien et al. [40]. Black square: N-acetylglucosamine; grey circle: mannose, black circle: glucose. The fragment ions are labelled according to the nomenclature of Domon and Costello [41]. [file 13007_2018_374_MOESM1_ESM.tif]

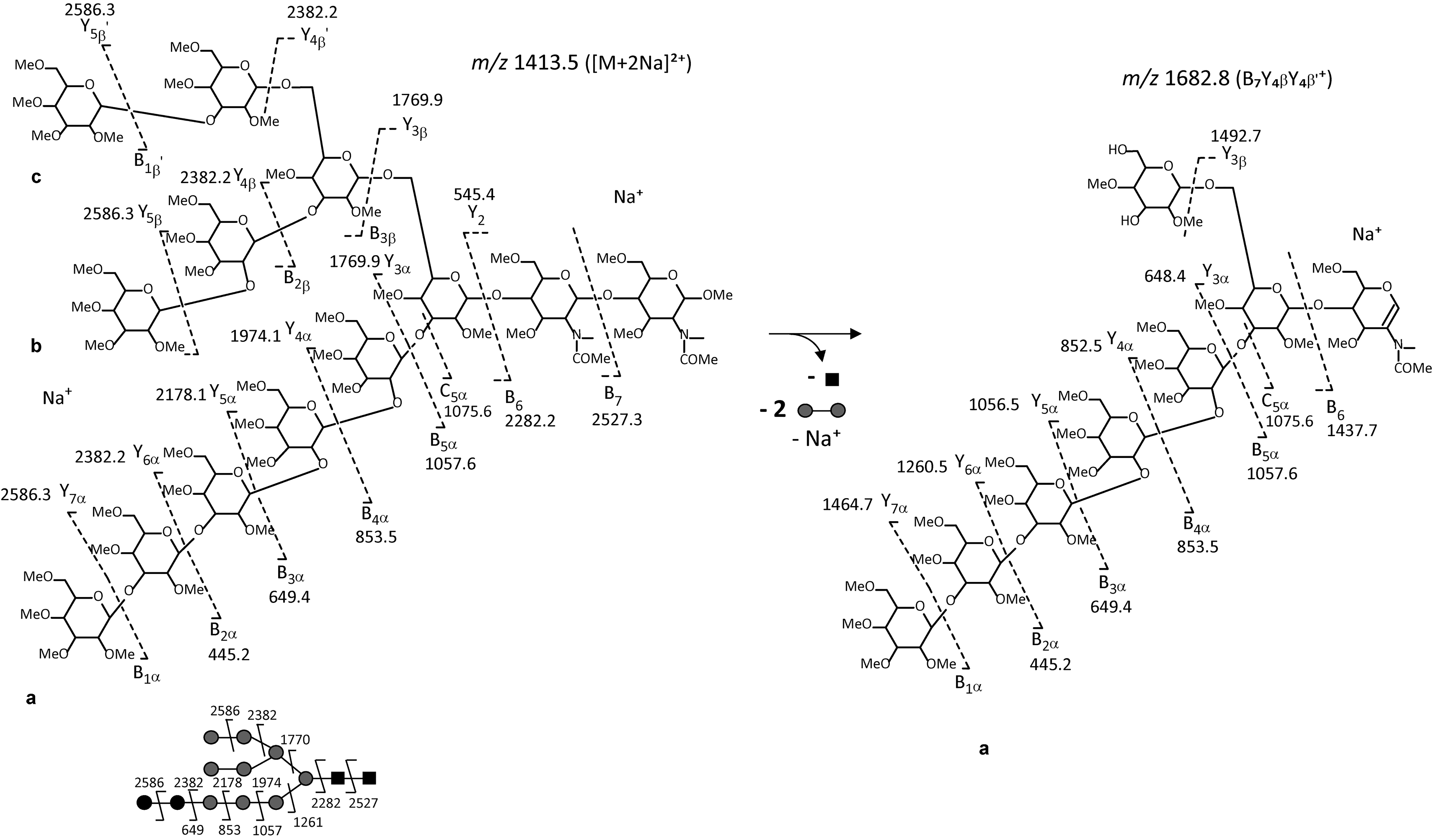

Supplement: Supplementary file 2 — Additional file 2. Scheme representing a fragmentation pathway complementary to the one depicted in Fig. 5. Cleavages of the glycosylic bond and cross ring cleavages are represented by dotted lines. The fragment ions are labelled according to the nomenclature of Domon and Costello [41]. [file 13007_2018_374_MOESM2_ESM.tif]

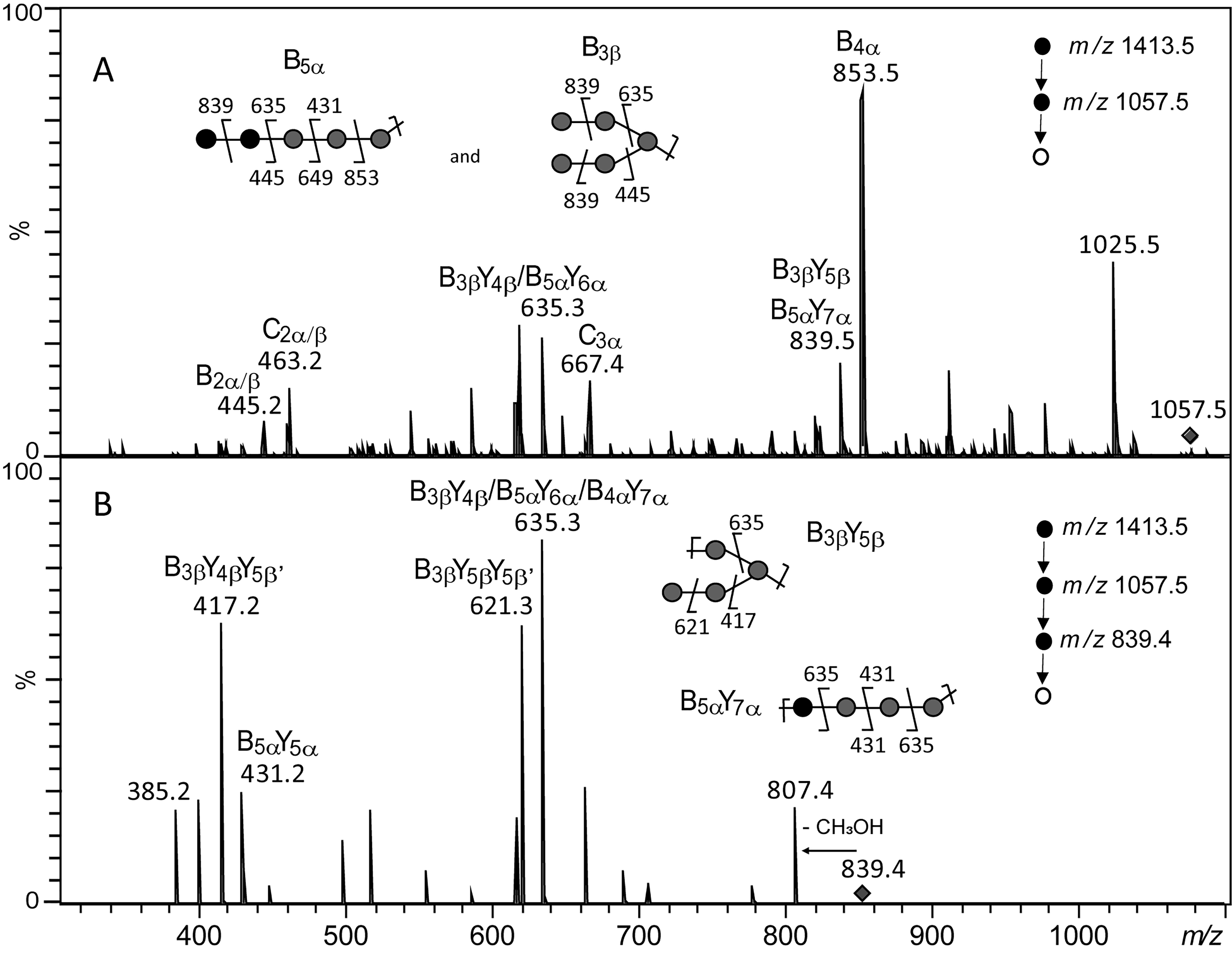

Supplement: Supplementary file 3 — Additional file 3. Multistage tandem mass spectrometry analyses of the structure of the LLO-released oligosaccharide isolated from P. tricornutum. ESI-MSn spectra with n = 3 (panel A) and n = 4 (panel B) of permethylated Hex11HexNAc2 derivative (m/z 1413.5 corresponding to [M + 2Na]2+ precursor ion) isolated from P. tricornutum. On each panel, the ion selected for the fragmentation analysis is shown with a diamond and its fragmentation pattern is proposed according to Prien et al. [40]. Black square: N-acetylglucosamine; grey circle: mannose, black circle: glucose. The fragment ions are labelled according to the nomenclature of Domon and Costello [41]. Note that loss of 204 u correspond to the elimination of a 1,2-; 1,3-; 1,4- or 1,6-linked hexose residue while loss of 218 u correspond to the elimination of a terminal hexose residue. Therefore, the successive losses of two 218 u (m/z 1057.5 → m/z 839.4 → m/z 621.3) indicate a di-antenna ion m/z 1057.5 while the successive losses of 204 u (m/z 1057.5 → m/z 853.4 → m/z 649.3) indicate a linear ion m/z 1057.5. [file 13007_2018_374_MOESM3_ESM.tif]

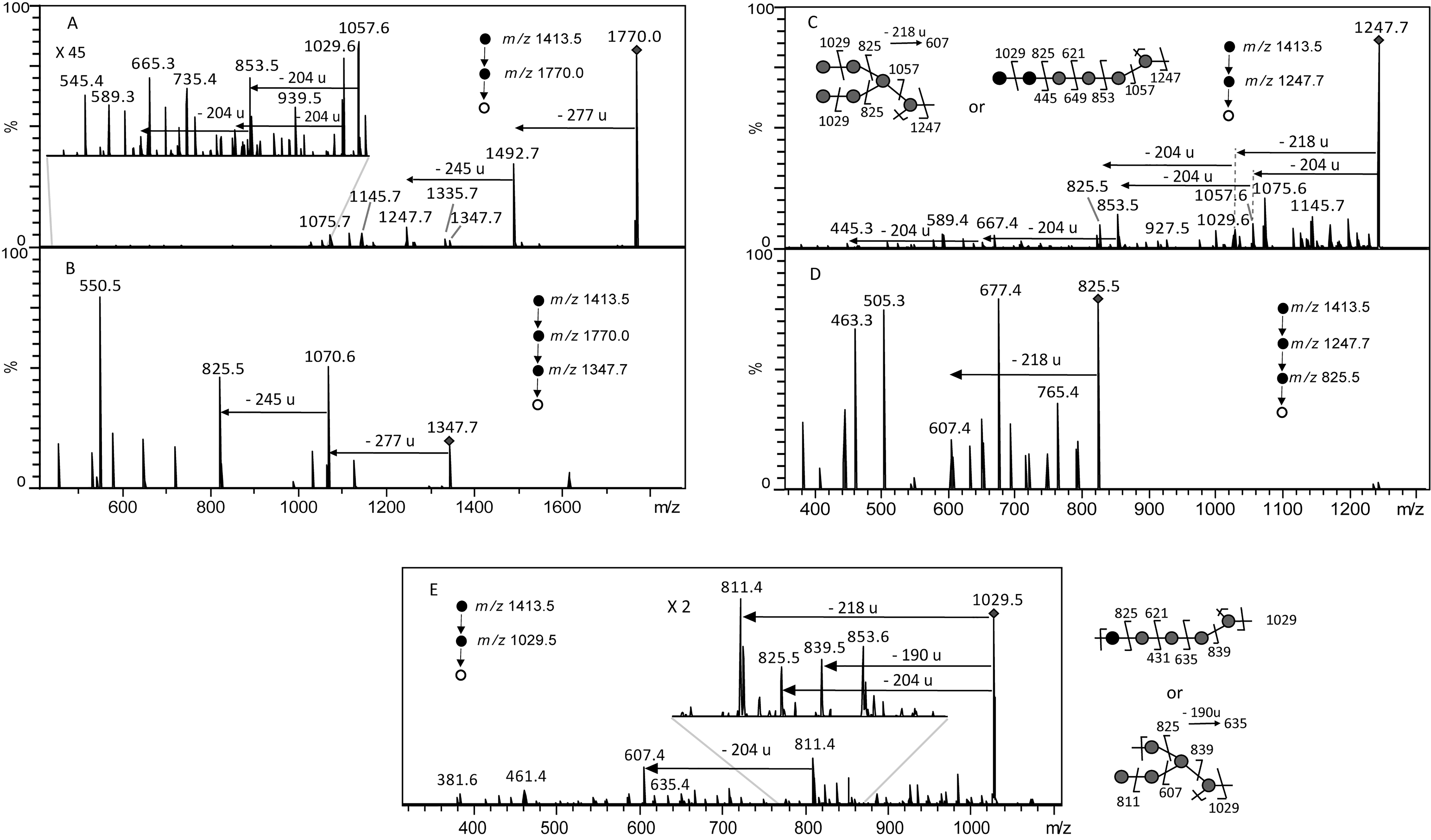

Supplement: Supplementary file 4 — Additional file 4. Multistage tandem mass spectrometry analyses of the structure of the LLO-released oligosaccharide isolated from P. tricornutum. ESI-MSn spectra with n = 3 (panels A, C and E) and n = 4 (panel B and D) of permethylated Hex11HexNAc2 derivative (m/z 1413.5 corresponding to [M + 2Na]2+ precursor ion) isolated from P. tricornutum. On each panel, the ion selected for the fragmentation analysis is shown with a diamond and its fragmentation pattern is proposed according to Prien et al. [40]. Black square: N-acetylglucosamine; grey circle: mannose, black circle: glucose. The fragment ions are labelled according to the nomenclature of Domon and Costello [41]. Note that loss of 190 u correspond to the elimination of a hexose residue which was linked with three other residues in the oligosaccharide (for example, a 1,3,6-linked hexose residue). Therefore, the successive losses of two 218 u (m/z 1247.6 → m/z 1029.5 → m/z 811.5) indicate a di-antenna ion m/z 1247.6 while the loss of 190 u followed by successive losses of 204 u (m/z 1247.6 → m/z 1057.5 → m/z 853.5 → m/z 649.4 → m/z 445.3) indicate a linear ion m/z 1247.6. [file 13007_2018_374_MOESM4_ESM.tif]
